# Multisite, External Validation of an AI-Enabled ECG Algorithm for Detection of Low Ejection Fraction

**DOI:** 10.1016/j.jacadv.2025.102537

**Published:** 2026-01-16

**Authors:** Rickey E. Carter, Patrick W. Johnson, Jordan B. Strom, Jonathan W. Waks, Andrew Krumerman, Kevin J. Ferrick, Roger DeRaad, Benjamin A. Steinberg, Mikolaj A. Wieczorek, Jessica Cruz, Zachi I. Attia, Francisco Lopez-Jimenez, Paul A. Friedman, Samir Awasthi, Mohan Krishna Ranganathan, Rakesh Barve, Heather M. Alger, Konstantinos C. Siontis, Peter A. Noseworthy

**Affiliations:** a Department of Quantitative Health Sciences, Mayo Clinic, Jacksonville, Florida, USA; b Division of Cardiovascular Medicine, Beth Israel Deaconess Medical Center, Boston, Massachusetts, USA; c Harvard-Thorndike Electrophysiology Institute, Beth Israel Deaconess Medical Center Havard Medical School, Boston, Massachusetts, USA; d Department of Cardiology Northwell Health, Mount Kisco, New York, USA; e Division of Cardiology, Montefiore Medical Center, New York, New York, USA; f Monument Health Clinical Research, Rapid City, South Dakota, USA; g Division of Cardiovascular Medicine, University of Utah, Utah, USA; h Department of Cardiovascular Medicine, Mayo Clinic, Rochester, Minnesota, USA; i Anumana, Inc, Cambridge, Massachusetts, USA

**Keywords:** external validation, left ventricular systolic dysfunction, software as a medical device

## Abstract

**Background:**

Low left ventricular ejection fraction (LEF) can progress undiagnosed. Artificial intelligence–based electrocardiogram (ECG-AI) screening may provide a scalable means to detect LEF.

**Objectives:**

The purpose of this study was to validate a complete ECG-AI software as a medical device for LEF detection.

**Methods:**

Four geographically diverse sites in the United States identified patients with both ECGs and transthoracic echocardiograms performed within 30 days of each other in clinical practice. Data were electronically extracted to specific guidelines and transmitted to the coordinating center for analysis.

**Results:**

Records of 16,000 subjects were extracted, resulting in an evaluable set of 13,960 subjects (mean age 66 years; 52% male). The device demonstrated excellent discrimination (AUROC: 0.92 [95% CI: 0.91-0.93]) and was 84.5% (95% CI: 82.2%-86.6%) sensitive and 83.6% (95% CI: 82.9%-84.2%) specific for LEF. The overall prevalence of LEF in the study data set was 7.9%, with LEF among 1.6% of the ECG-AI negative and 30.5% of ECG-AI positive subjects, contributing to positive and negative predictive values of 30.5% (95% CI: 28.8%-32.1%) and 98.4% (95% CI: 98.2%-98.7%), respectively.

**Conclusions:**

External validation studies such as this one provide a rigorous framework to validate an algorithm’s performance. This study demonstrated the algorithm’s strong diagnostic accuracy over a geographically diverse, independent set of patients. In this generally unselected population, the algorithm produced a test negative result in 78% of the cases, suggesting potential utility as a rule-out strategy to defer echocardiography when other clinical findings are absent.

Left ventricular systolic dysfunction (LVSD) from any etiology has been estimated recently to be present in 7.3% of the general population.^1^ While LVSD can be initially asymptomatic in many patients, it is typically associated with progressive symptoms of heart failure (HF) and an adverse prognosis if unrecognized and left untreated. Low left ventricular ejection fraction (LEF) is an indicator of LVSD progression toward symptomatic HF.^2^ There are well-studied, effective treatments that can reduce symptoms, prevent progression, and prolong survival when initiated early in the disease course.^3^^,^^4^ As such, early detection of LVSD is critical for timely diagnostic investigation and initiation of effective therapies. Current methods to screen for LEF require cardiovascular imaging, which may be expensive, not readily available, or time-consuming to be implemented at scale.^5^

The advent and widespread success of deep learning artificial intelligence (AI) algorithms applied to digital electrocardiogram (ECG) waveforms has the potential to address the barriers for testing. An AI-enhanced ECG (ECG-AI) interpretation algorithm was previously developed for the detection of LEF from the 12-lead ECG at a single large tertiary care institution.^6^ This algorithm demonstrated validity when it was retrospectively applied to various clinical scenarios and when it was tested in a prospective implementation trial at the same institution.^7^ However, external validation of such algorithms in independent geographically and demographically diverse populations is essential to ensure their robustness.^8^, ^9^, ^10^ This type of multicenter validation study is also necessary to support the transition from an innovative investigational tool to the wider use of the technology as a regulated software as a medical device (SaMD) that is allowed for use in routine clinical care settings.^11^, ^12^, ^13^ The objective of this study was to establish the diagnostic performance of the fully matured 12-lead ECG-AI algorithm SaMD in detecting LEF in a geographically diverse, independent set of patients in the United States.

## Methods

### Study design and site selection

This multisite, retrospective study (A Multi-Center Study of Detection of Low Ventricular Ejection Fraction; NCT04963218) was designed as an external validation study of an established, locked algorithm and conducted after approval by the Mayo Clinic Institutional Review Board (submitted 5/12/2021; approved 5/27/2021). Sites, which were required by regulatory input to be distinct of the primary training sites (ie, Mayo Clinic), were selected to be as representative of the national population of the United States as possible. Secondarily to the geographic representation, sites were also assessed on the ability to electronically extract the digital ECG waveforms and required clinical data from their medical records. Finally, the clinical protocols for reading the transthoracic echocardiogram were reviewed to ensure that the extracted left ventricular ejection fraction (LVEF) measurements would be considered reliable and consistent with the use of the data for regulatory approval. Participating sites were provided a detailed data extraction technical guide to define the approach to retrospective data retrieval. The digital ECG files were shared in a secure and deidentified fashion with the coordinating institution (Mayo Clinic) along with the LVEF value and basic demographic and clinical characteristics per patient. Study materials and data will be made available as specified in the data sharing statement accompanying this publication.

### Participants

Only patients aged at least 18 years at the time of qualifying echocardiogram and ECG were eligible for inclusion in the study. All selected patient records were required to have at least one qualifying digital 12-lead ECG paired with an echocardiogram with quantitative LVEF information within 30 days of the date of the ECG. The protocol specified the use of Simpson’s biplane method of discs for the quantitative assessment of LVEF; visual estimation of LVEF was not permitted.^14^^,^^15^ The protocol did not exclude patient records based on any history of cardiac disease. Digital ECGs were required to meet a set of quality assurance checks to be deemed eligible for inclusion in this study. Briefly, ECGs were required to be at least 10 seconds in length and reported at 500 Hz. Furthermore, ECGs would be screened for absent lead data (zero modulation of ECG on at least one channel) and noise/electrical interference that persisted after filtering. The original training data set for development of the ECG-AI model excluded paced ECGs, and therefore, paced ECGs were ineligible for inclusion in this multicenter validation study. There were no restrictions applied to the selection of cases based on cardiac diagnoses, including conduction disorders, that may have impacted the ECG. Patient records were extracted so that each patient would only supply one observation for the final analysis.

### Investigational device

The experimental low ejection fraction ECG-AI algorithm was deemed an investigational medical device used for clinical decision support. This SaMD was designed to detect whether a patient has an LVEF ≤ 40% based on only the input of the digital data from an ECG. The algorithm, which was built upon the original Mayo Clinic-developed algorithm,^6^ uses a convolutional neural network to interpret the digital 12-lead ECG data. The only input into the SaMD was the digital ECG (XML format) sampled at 500 Hz; no other clinical data are used as inputs into the model.

The original algorithm was implemented using TensorFlow and trained to classify if a patient has LVEF ≤35% vs >35% with a reported sensitivity, specificity, and area under the receiving operator characteristic curve (AUROC) of 86.3%, 85.7%, and 0.93, respectively.^6^ For the algorithm used within the SaMD, a similar neural network architecture was implemented using PyTorch, which is another flexible, open-source, computational framework used to support deep learning models. In the process of adapting the algorithm to this new computational framework, the model was optimized to classify if a patient has LVEF ≤40% vs >40% to align with the diagnostic threshold established in current clinical practice guidelines for the management of HF.^2^ In addition, the SaMD incorporated several design features such as quality checks on the input to identify and reject low-quality ECGs and signal processing to remove noise artifacts prior to presenting the ECG to the ECG-AI neural network model. The SaMD algorithm was designed to be self-contained to support a wide range deployment scenarios and clinical decision support systems.

As this SaMD was intended to be a finished medical device, quality assurance checks on the input were included to ensure they met specifications sufficient to return a prediction (Supplemental Figure 1). As a SaMD, ECG-AI LEF is required to meet specifications described in 21 CFR 870.2380, which currently restricts ECG-AI algorithm results to be provided as a categorical classification (eg, binary) output rather than a continuous score as the output to support clinical judgment.

### Reference criterion

The reference criterion for the study was the LVEF estimated from a transthoracic echocardiogram as reported in the medical record (ie, the physician-adjudicated value reported by the original read of the echocardiogram). Only LVEF values obtained with the Simpson’s biplane method were selected. Given the retrospective nature of the study and the scale at which data were to be gathered, no independent, prospectively applied adjudication of the reported LVEF values was performed. Instead, and as part of the site qualification process, site-specific Manual of Operations and Standard Operating Procedures for calculating the ejection fraction were reviewed. Only sites that passed this quality review were considered for this study.

### Statistical analysis

The primary hypotheses were based on the sensitivity and specificity for the detection of LEF (ie, LVEF ≤40%) using only the AI model output. Specifically, sensitivity and specificity each were required to exceed 0.80 (80%). The alternative hypotheses were stated as 2-sided tests at alpha = 0.05; however, the overall acceptance criteria of the study required that both of the lower bounds for the respective 95% exact CIs exceed the null values of 80%.

The sample size estimation for this study was based on a null value for sensitivity of 0.80 (80%) since the prevalence of LEF in the sample was assumed to be in the range of 5% to 10%. The algorithm’s performance, for both sensitivity and specificity, was hypothesized to be at least 0.85 based on prior validation studies.^6^^,^^7^ The level of significance and type II error rate were set at 0.05 (2-sided) and 0.10 (90% power), respectively. This resulted in an initial sample size of 617 cases with LEF using the calculation routine for a one-sample, exact test for a proportion as programmed into PASS version 14. The overall sample size was determined by scaling the total sample size over the range of prevalence values that were considered plausible (ie, 5% to 10%). At a ratio of 1:9 for LEF, a total sample size of 6,170 would have been required. With a prevalence of 5% (1:19), the estimated sample size increased to 12,340. Given the uncertainty with the prevalence and an unknown number of nonconformant ECGs that would be extracted, a total sample size of 16,000 was selected. Four clinical sites would each be expected to extract up to 4,000 unique, eligible medical records.

For the primary analysis, the continuous LVEF reported by the sites was categorized into LEF (“disease positive;” LVEF ≤40%) or not (“disease negative;” LVEF >40%). Upon analysis of the input ECGs, the SaMD reported a binary prediction for each ECG (ie, a positive test result for LVEF ≤40% or a negative test result for LVEF >40%), or an error. The binary prediction was based on a threshold selected prior to the algorithm’s use in this trial and was not adjusted based on data collected during this external validation study. The cross tabulation of the device output with LEF status determined the device’s sensitivity and specificity. To facilitate hypothesis testing and statements on precision of estimated effects, 95% exact CIs were computed. The overall acceptance criterion for the study required both of the lower limits to exceed 0.80 (equivalent to a one-sided, exact binomial tests to a null value of 0.8 vs an alternative >0.80 using an alpha = 0.025 level of significance).

Other measures of diagnostic performance were assessed as secondary endpoints and were summarized similarly as point estimates and 95% CIs. AUROC and area under the precision-recall curve (AUCPR) were generated using the continuous model output to describe overall model performance. The AUCPR plots the precision (positive predictive value) relative to the recall (sensitivity) over a range of thresholds to provide an overall measure of diagnostic performance of a binary classifier with imbalanced data (ie, low prevalence of LEF). No formal hypothesis testing of these measures was proposed in the protocol or Statistical Analysis Plan. Negative and positive predictive values were calculated on observed sample data (ie, at the prevalence observed in the data retrieved). Predefined subgroup analyses were conducted. Breslow-Day tests for homogeneity of the OR were computed to summarize differential performance, if any, of the device among subgroups. Regularized expressions and other text processing approaches were used to determine conduction disorders present on the ECGs based on the diagnosis text stored in the digital ECG files. Not all sites provided the full XML file to support this analysis.

Sensitivity analyses were conducted to examine the percentage of test positive ECG screenings over the range of LVEF values observed in the study. In addition, an intention to diagnose analysis was generated that conservatively considered instances where the device prediction was not made (index test not available according to STARD [Standards for Reporting Diagnostic Accuracy] criteria) as either false negatives or false positives for cases that were true LEF or not based on the reference criterion.^16^ No correction to the type I error rates was applied to the estimated CIs. Statistical analyses were conducted using R version 4.0.3.

### Role of the funding source

Mayo Clinic served as the lead clinical and data coordination center for this study. Funding to support the trial was provided by Anumana, Inc, as a contract to Mayo Clinic. Mayo Clinic had exclusive access to the data. The sponsor did not participate in data analysis. Summary reports, including the clinical studies report required for a Food and Drug Administration (FDA) filing, were drafted jointly by Mayo Clinic and Anumana, Inc.

## Results

**Figure 1:**
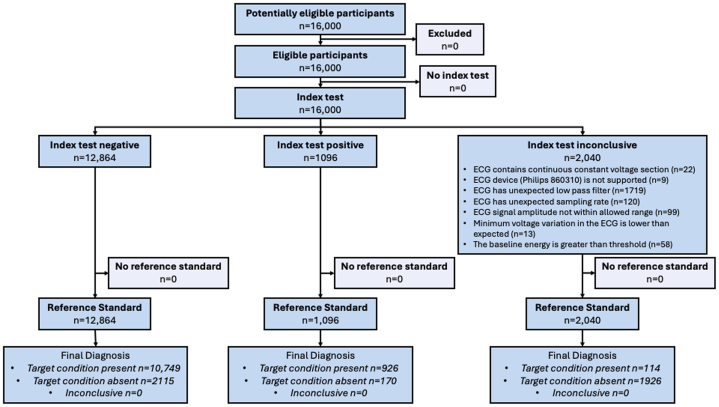
**Study Flowchart** A total of 16,000 heath records across 4 sites were electronically retrieved and deidentified for the study. Most of the (1,719/2,040) exclusions for the study were related to the incompatibility of the digital ECG waveforms with the investigational device, specifically the low pass filter configuration of the machine at the time of ECG acquisition. The final analysis set consisted of 13,960 health records. ECG = electrocardiogram.

A total of 16,000 records derived from 16,000 unique patients were extracted from 4 institutions across the United States (Beth Israel Deaconess Medical Center, Boston, Massachusetts, USA; Montefiore Medical Center, New York, New York, USA; Monument Health, Rapid City, South Dakota, USA; University of Utah, Salt Lake City, Utah, USA) (Central Illustration). Figure 1 presents the disposition of the records in the context of the final analysis. ECGs from 3 of the study sites (Beth Israel Deaconess Medical Center, Montefiore Medical Center, University of Utah) were collected on the General Electric (GE) ECG platform; ECGs from Montefiore Medical Center were collected on the Philips ECG platform. A total of 2,040 records were eliminated because they failed ECG quality checks and thus were incompatible with the investigational device. The primary analysis was conducted on the 13,960 conformant ECGs in which a total of 1,096 (7.9%) patients were identified as having LEF (ie, LVEF ≤40%). The prevalence of LEF varied among the sites with a range of 5.6% (151/2,717) for Montefiore to 8.3% (320/3,873) for Monument Health. The characteristics of the analyzed cohort are presented in Table 1. Briefly, the mean (range) age was 66 (18-105) years, 52% of the sample was male, and 66% were White. The racial distribution varied by site as intended in the design of the study. Supplemental Table 1 shows the demographic profile of the participants by LVEF classification.

**Central Illustration:**
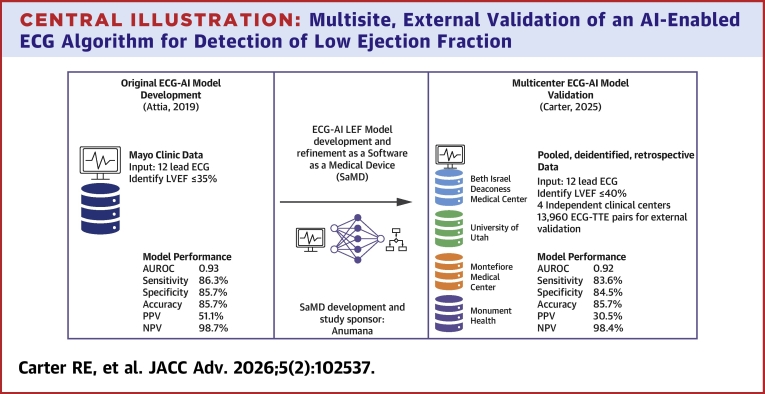
**Multisite, External Validation of an AI-Enabled ECG Algorithm for Detection of Low Ejection Fraction** This summarizes the organization structure of the external validation study along with the central findings of the study. AI = artificial intelligence; AUCPR = area under the precision-recall curve; AUROC = area under the receiving operator characteristic curve; ECG = electrocardiogram; LVEF = left ventricular ejection fraction; NPV = negative predictive value; PPV = positive predictive value; TTE = transthoracic echocardiogram.

**Table 1:** Sample Characteristics by Site

	Total^a^ (N = 13,960)	Beth Israel Deaconess Medical Center (n = 3,968)	Montefiore Medical Center (n = 2,717)	Monument Health (n = 3,873)	Utah (n = 3,402)
Age, y	66.0 (53.0, 76.6)	66.4 (53.3, 77.1)	67.0 (56.0, 78.0)	69.0 (58.0, 80.0)	60.2 (44.8, 71.4)
Age group, y					
<40	1,619 (11.6%)	492 (12.4%)	246 (9.1%)	246 (6.4%)	635 (18.7%)
40 49	1,280 (9.2%)	349 (8.8%)	211 (7.8%)	280 (7.2%)	440 (12.9%)
50 59	2,199 (15.8%)	601 (15.1%)	446 (16.4%)	545 (14.1%)	607 (17.8%)
60 69	3,188 (22.8%)	860 (21.7%)	624 (23.0%)	932 (24.1%)	772 (22.7%)
70 79	3,019 (21.6%)	913 (23.0%)	571 (21.0%)	898 (23.2%)	637 (18.7%)
≥80	2,655 (19.0%)	753 (19.0%)	619 (22.8%)	972 (25.1%)	311 (9.1%)
Male	7,251 (52.0%)	2097 (52.8%)	1,226 (45.1%)	2053 (53.0%)	1875 (55.1%)
Height, m	1.7 (1.6, 1.8)	1.7 (1.6, 1.8)	1.7 (1.6, 1.7)	1.7 (1.6, 1.8)	1.7 (1.6, 1.8)
Weight, kg	80.5 (67.1, 96.2)	78.5 (65.8, 93.0)	76.8 (65.4, 91.0)	81.6 (66.6, 97.6)	85.0 (71.2, 101.4)
BMI	27.8 (24.0, 32.6)	27.4 (23.9, 31.9)	27.8 (24.0, 32.3)	27.9 (23.7, 33.1)	28.3 (24.5, 33.4)
Weight status					
Underweight	422 (3.4%)	106 (2.7%)	65 (3.1%)	196 (5.1%)	55 (2.2%)
Normal weight	3,457 (28.0%)	1,203 (30.4%)	589 (28.2%)	1,045 (27.2%)	620 (25.3%)
Overweight	3,907 (31.7%)	1,320 (33.4%)	694 (33.2%)	1,114 (29.0%)	779 (31.8%)
Obese	4,542 (36.8%)	1,325 (33.5%)	743 (35.5%)	1,482 (38.6%)	992 (40.6%)
White	8,944 (65.9%)	2,353 (59.3%)	694 (25.5%)	3,129 (80.8%)	2,768 (91.7%)
Black or African American	1,371 (10.1%)	476 (12.0%)	792 (29.1%)	23 (0.6%)	80 (2.7%)
American Indian or Alaska Native	716 (5.3%)	17 (0.4%)	2 (0.1%)	637 (16.4%)	60 (2.0%)
Asian	300 (2.2%)	155 (3.9%)	69 (2.5%)	14 (0.4%)	62 (2.1%)
Native Hawaiian or other Pacific Islander	55 (0.4%)	3 (0.1%)	1 (0.0%)	3 (0.1%)	48 (1.6%)
Hispanic	1,475 (11.0%)	294 (8.3%)	836 (30.8%)	52 (1.3%)	293 (9.0%)
Hypertension	8,338 (59.7%)	2,768 (69.8%)	2090 (76.9%)	1,402 (36.2%)	2078 (61.1%)
Heart failure	3,391 (24.3%)	1,266 (31.9%)	914 (33.6%)	440 (11.4%)	771 (22.7%)
Coronary revascularization	1,440 (10.3%)	522 (13.2%)	398 (14.6%)	203 (5.2%)	317 (9.3%)
Myocardial infarction	2,450 (17.6%)	759 (19.1%)	474 (17.4%)	339 (8.8%)	878 (25.8%)
Alcoholism	1,178 (8.4%)	447 (11.3%)	311 (11.4%)	162 (4.2%)	258 (7.6%)
Diabetes mellitus	3,793 (27.2%)	1,250 (31.5%)	1,200 (44.2%)	467 (12.1%)	876 (25.7%)
Rheumatic fever	5 (0.0%)	2 (0.1%)	2 (0.1%)	0 (0.0%)	1 (0.0%)
Days from ECG to echocardiogram	1.0 (1.0, 3.5)	1.0 (0.0, 3.0)	2.0 (1.0, 4.0)	1.0 (1.0, 2.0)	1.1 (0.4, 6.8)
Conduction disorder^b^	1,518 (15.2%)	Not determined	381 (14.0%)	686 (17.7%)	451 (13.3%)
RBBB	681 (6.8%)	—	202 (7.4%)	248 (6.4%)	231 (6.8%)
LBBB	354 (3.5%)	—	69 (2.5%)	195 (5.0%)	90 (2.6%)
LPFB	35 (0.4%)	—	7 (0.3%)	19 (0.5%)	9 (0.3%)
LAFB	239 (2.4%)	—	62 (2.3%)	110 (2.8%)	67 (2.0%)
BFB	209 (2.1%)	—	41 (1.5%)	114 (2.9%)	54 (1.6%)
LVEF ≤40%	1,096 (7.9%)	383 (9.7%)	151 (5.6%)	320 (8.3%)	242 (7.1%)
LVEF	62.0 (56.0, 67.0)	62.0 (56.0, 68.0)	62.0 (56.0, 67.0)	61.0 (55.0, 66.0)	61.8 (55.6, 67.3)

BFB = bifascicular block; BMI = body mass index; ECG = electrocardiogram; LAFB = left anterior fascicular block; LBBB = left bundled branch block; LPFB = left posterior fascicular block; LVEF = left ventricular ejection fraction; RBBB = right bundle branch block.

Values are median (Q1, Q3) or n (%).

a Missing data are excluded from all calculations. The following is a summary of the missing data for the overall variable along with the sample size by site using the format (BIDMC:MONTEFIORE:MONUMENT:UTAH). Sex (Nmiss = 3 [0:1:0:2]), height (Nmiss = 1,578 [12:622:36:908]), weight (Nmiss = 1,093 [12:626:36:419]), BMI (Nmiss = 1,632 [14:626:36:956]), weight status (Nmiss = 1,632 [14:626:36:956]), White (Nmiss = 384 [0:0:0:384]), Black or African American (Nmiss = 384 [0:0:0:384]), American Indian or Alaska Native (Nmiss = 384 [0:0:0:384]), Asian (Nmiss = 384 [0:0:0:384]), Native Hawaiian or other Pacific Islander (Nmiss = 384 [0:0:0:384]), Hispanic (Nmiss = 591 [432:0:0:159]), conduction disorder (Nmiss = 3,968 [3,968:0:0:0]), right bundle branch block (Nmiss = 3,968 [3,968:0:0:0]), left bundled branch block (Nmiss = 3,968 [3,968:0:0:0]), left posterior fascicular block (Nmiss = 3,968 [3,968:0:0:0]), left anterior fascicular block (Nmiss = 3,968 [3,968:0:0:0]), and bifascicular block (Nmiss = 3,968 [3,968:0:0:0]).

b Any conduction disorder is defined as a case having any of the following conduction disorders: RBBB, LBBB, LPFB, LAFB, or BFB. Diagnostic statements required to classify ECGs as having a conduction disorder were not extracted as a part of the original data collection for BIDMC.

**Figure 2:**
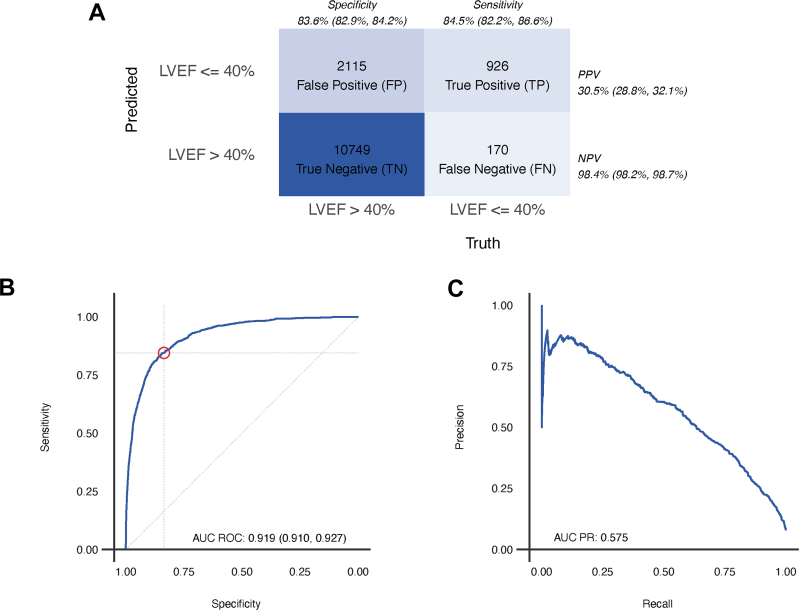
**Summary of Diagnostic Performance ECG-AI Algorithm Results** (A) The confusion matrix using the FDA-cleared binary model output. (B, C) The receiver-operating characteristics curve and precision-recall curve, along with associated areas under the respective curve, using the continuous model output that is available with the device in a research setting. The 95% CIs presented in the confusion matrix are exact binomial CIs. The receiver-operating characteristics curve and precision-recall curve were generated using the continuous model output. The CI for AUC is based on the Delong method. AI = artificial intelligence; AUCPR = area under the precision-recall curve; AUROC = area under the receiving operator characteristic curve; FDA = Food and Drug Administration; LVEF = left ventricular ejection fraction; NPV = negative predictive value; PPV = positive predictive value.

The device achieved sensitivity and specificity of 84.5% (95% CI: 82.2%-86.6%) and 83.6% (95% CI: 82.9%-84.2%), respectively, for the detection of LEF (Figure 2A). Positive and negative predictive values of the device’s primary binary classifier at the observed prevalence were 30.5% (95% CI: 28.8%-32.1%) and 98.4% (95% CI: 98.2%-98.7%), respectively. The likelihood ratio test positive and negative values were 5.14 (95% CI: 4.91-5.38) and 0.19 (95% CI: 0.16-0.21). These values, when combined, resulted in a diagnostic OR of 27.7 (95% CI: 23.4-32.8). The overall discrimination of the model was high with an AUROC of 0.919 (95% CI: 0.910-0.927) and a corresponding AUCPR of 0.575 (Figures 2B and 2C). The intention to diagnosis analysis, an analysis that considered all inconclusive AI screenings (n = 2,040, Figure 1) as incorrect test, sensitivity and specificity remained robust at 76.5% (95% CI: 74.0%-78.9%) and 72.7% (95% CI: 72.0%-73.4%), respectively (Supplemental Table 2).

**Figure 3:**
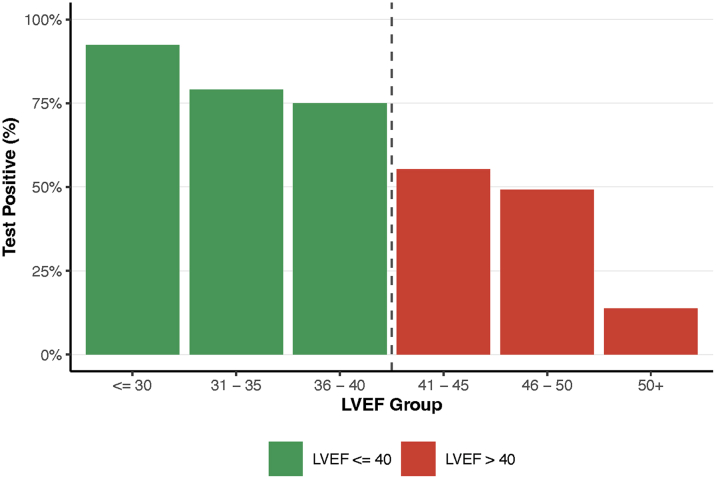
**Test Positive Percentage by Ejection Fraction** This plot summarizes the percentage of ECGs classified as test positive over the range of ejection fraction values observed. The dashed line at LVEF 40% represents the algorithms internal definition of LVSD. For cases with ejection fraction below this threshold, the percentage test positive is interpretable as positive predictive value. Likewise, for tests that were positive above this limit, they would represent false positive predictions. LVSD = left ventricular systolic dysfunction; other abbreviation as in Figure 2.

To assess the model performance over a range of disease severity, the percentage of ECGs resulting in a positive prediction (test positive) was plotted over a range of LVEFs observed in the study (Figure 3). This analysis demonstrated a monotonic relationship of disease severity, as defined by the LVEF classification, and the number of ECGs that were classified as test positive. In particular, for severe LVSD (LVEF ≤30%), the percentage of cases resulting in a positive AI prediction was 92.3% (503/545). For patients with normal ejection fractions (LVEF ≥50%), this percentage was only 13.7% (1,638/11,940). Supplemental Table 3 presents a tabulation of the device’s correct predictions (true positive, true negative) and incorrect predictions (false positive, false negative) by clinical and study characteristics.

Diagnostic performance did not significantly vary by site with sensitivity and specificity both exceeding 80% for all 4 sites (Breslow-Day *P* = 0.45, Supplemental Table 4). Supplemental Figures 2 to 6 display the subgroup analyses by sex and age, race, medical history, conduction disorders, and ECG device manufacturer. No heterogeneity of performance was identified across sex, race, or ECG device manufacturer subgroups. The prevalence of a conduction disorder was 15.2% (1,518/9,992; n = 3,968 ECGs had the diagnostic text removed during the deidentification process). The SaMD also showed consistent performance in the presence of any conduction disorder and over specific types of conduction disorder (Supplemental Figure 5). However, there were differences in performance across certain subgroups defined by patient characteristics. The discrimination of the algorithm varied demonstrating lower performance in the extreme ages (OR 25.8 in patients <40 years, OR 16.3 in patients ≥80 years) as opposed to OR 43.4 in patients aged 50 to 59 years (*P* < 0.01, Supplemental Figure 2). This U-shaped distribution of performance aligns with low sensitivity in the <40 years age group and lower specificity in the oldest groups. Furthermore, device performance was inferior in those with a clinical history of HF or myocardial infarction as compared to those without such history (*P* < 0.01 for both sets of subgroups). Similarly, history of coronary revascularization showed some evidence for heterogeneity in device performance (Breslow-Day test, *P* = 0.07). In each of these subgroups, LEF prevalence was high (>17% for the 3 conditions) and the algorithm had high sensitivity and low specificity. This combination, at the higher prevalence values, resulted in increased positive predictive value (>38%) and negative predictive value (>94%).

## Discussion

This study was a multicenter, external validation of an ECG-AI algorithm for the detection of LEF originally developed at Mayo Clinic and matured and refined by Anumana into a software medical device that has received regulatory clearance for use in routine clinical practice. To ensure robustness and generalizability of this study’s findings, geographically diverse sites were selected to create a validation data set generally representative of the U.S. population. The SaMD met the predefined acceptance criteria by achieving lower values of the 95% CIs of both sensitivity and specificity exceeding 80% for the detection of LEF from the 12-lead ECG waveforms alone.

Positive and negative predictive values were also evaluated as secondary/exploratory endpoints in this study in the overall sample and across subgroups. The overall values for positive predictive value (PPV) and negative predictive value (NPV) were 30.5% and 98.4%, respectively. With an LEF prevalence of 8% in the study data set, the PPV was lower than NPV as expected and consistent with the intended use as a screening tool to identify otherwise asymptomatic but at-risk patients who may benefit from additional screening by echocardiography for early detection. The high value for NPV is consistent with using the test to rule-out patients who may not need additional screening, in the absence of other risk factors. This study highlights the potential of AI-enhanced ECG screening as a scalable screening tool for early detection of reduced LVEF, addressing an unmet clinical need and enabling interventions to reduce risk of developing HF. The current guidelines recommend the use of biomarkers for risk stratification and initial diagnosis for patients presenting with symptoms, such as shortness of breath^2^; ECG-AI could provide a low-cost, high-access solution for screening of Stage A patients who have risk factors and may benefit from additional confirmatory diagnosis with echocardiography and primary prevention.

External validation is a prerequisite in the translation of deep learning AI solutions into practice. The underlying innovation of using convolutional neural networks, or more generally deep learning, for the detection of LVSD from the 12-lead ECG has been retrospectively validated in various clinical settings in Mayo Clinic cohorts by the original investigators.^7^^,^^17^ Furthermore, in a prospective, nonrandomized study of over 22,000 ECGs obtained as part of routine care in the same institution, implementation of the earlier version of the ECG-AI algorithm led to an increase in diagnosis of low EF.^18^ While the validity of that algorithm has been previously tested in an external population-based study from Russia with a low prevalence of LVSD (0.6%),^19^ the current study represents the first external validation assessment of the fully matured and marketable version of the ECG-AI algorithm in a multicenter US-based population derived from diverse clinical practices. In the study population, LVSD prevalence more closely mirrored the prevalence of LVSD anticipated in patients undergoing clinically indicated cardiac testing.^1^ It should also be emphasized that algorithm performance was not impacted by the various ECG device models included in this study. These data demonstrate that application of an ECG-AI algorithm for the detection of LVSD can be performed with high accuracy in diverse clinical settings using digital, 12-lead ECG waveforms. Recent data have also highlighted the feasibility of applying adapted versions of this algorithm to patient-acquired smartwatch ECG tracings, though this approach requires more extensive validation.^20^

The performance of the algorithm was mostly uniform across predefined subgroups according to demographic, clinical, and ECG characteristics. Supplemental Table 3 showed that among the more medically complex patients with comorbidities, the device error more on the false positives than false negatives. Nonetheless, we also observed variation in performance over age groups and lower performance in patients with a clinical history of HF and those with prior myocardial infarction. The exact reasons underlying these observations are not fully known and require further testing. A high burden of cardiac and noncardiac comorbidities may confound the ECG-AI signature of low LVEF in patients in the extremes of the age. Furthermore, it is possible that ECG changes (beyond those assessed in subgroup analyses) in those with known HF or prior myocardial infarction may be associated with lower algorithm performance. Nonetheless, in the presence of such obvious ECG abnormalities further testing for the assessment of LV function is often clinically indicated regardless of ECG-AI results in the appropriate clinical context. Noteworthy, the prevalence of clinical HF and prior myocardial infarction in the algorithm’s training set were only 20% and 13%, respectively.^6^

Importantly, validation and replication of the approach has now been completed by others, and the methodology has been extended to cover many other cardiomyopathies, conduction disorders, and disease settings.^21^, ^22^, ^23^, ^24^ Thus, while the explainability of how the convolutional networks extract features from the digital ECGs remains an area of ongoing study, these algorithms are showing promising clinical utility as demonstrated in prospective clinical studies featuring ECG-AI algorithms.^18^^,^^25^ Regulatory clearance of algorithms is essential for widespread realized benefit from the emerging technologies such as AI-interpreted ECGs, and to the benefit of the populations, regulatory agencies have shown agility and acceptance of the evolving concepts of medical devices.^26^^,^^27^ The results of this external validation study were used in the regulatory submission that sought and received FDA clearance for the LVSD detection algorithm.

The study had several strengths and demonstrated design features that may be useful for future external validation studies. First, the data were extracted and rendered protocol-compliant by investigators at each participating sites. In this way, data deidentification practices were allowed to be site-specific and the extraction guide focused on only essential data elements to further maximize patient confidentiality. Next, the study engaged 3 sites that used GE-based ECG machines and one site using Philips-based ECG machines to provide greater exposure to ECGs machines used in practice. The study recruited a considerable number of American Indian participants (n = 716) as this population has been traditionally under-represented in clinical research.^28^ Variations in performance of deep learning ECG modeling for incident HF across racial groups have been recently recognized.^29^ In the current external validation, algorithm performance for detection of LVSD was homogeneous across racial subgroups.

### Study Limitations

Our study and underlying protocol have limitations. First, the protocol was written before study sites were selected, so it was not possible to reliably estimate the prevalence of LEF that would be observed in the final analysis set. To address this, a range of prevalence values was used in the sample size calculations to account for this uncertainty. Additionally, we needed to exclude paced ECGs as they were not eligible for the SaMD. Finally, the study was designed to evaluate the device performance in a real-world setting, so the protocol did not independently adjudicate the LVEF measurements. In this regard, the final clinical report values were used as the reference criterion, which is an approach consistent with the use of real-world data; however, it is known that LVEF estimation in centralized laboratories typically provides enhanced reliability relative to site-based measurements.^30^ The study sites also had various degrees of anonymization required by their institutional practices. This results in missing data across many clinical characteristics that limited the sample size for many of the subgroup analyses.

Next, there were inherent limitations in the device. The algorithm within the SaMD was developed on unpaced ECGs, so we are not able to estimate performance of the device on this important set of patients. Furthermore, as requested by the regulatory agency, was to only report a binary classification for test positive or negative. Thus, the prespecified endpoints for the study focused on the test performance based on the categorized device output. As part of this article, the area under the receiver operating characteristics curve and the AUCPR were added post hoc to better describe the model performance over a range of possible thresholds. While this study conducted robust external validation across 4 sites, there are many ECG devices that are in current practice that could not be studied. Thus, while the set of ECG machines that were utilized in the study was more comprehensive than the original development and validation studies, there were a limited number of Philips models available for testing. Additional studies on a larger array of machines, particularly from a world-wide perspective, will be required. Finally, the algorithm had to be version locked prior to conducting the final analysis—effectively guaranteeing that the device would consistently deliver the same output for the same input over time. While it is recognized that having dynamic updates to devices may yield enhanced performance,^30^ adapting and evolving the algorithm based on the data accrued in this study was not considered. This focus on rigor and reproducibility of output limits the ability to conduct exploratory analyses commonly associated with the initial development AI devices in medicine.^11^, ^12^, ^13^

In summary, the ECG-AI LEF SaMD tested in this multicenter, external validation study demonstrated diagnostic performance over a range of patient profiles, geographic sites, and device models that exceeded the protocol-specified measures of performance. This independent validation further establishes the utility of AI-based interpretation of the ECG to advance cardiovascular screening. However, additional studies focused on implementation are needed to advance our understanding on how to best include ECG-AI interpretation in their routine screening protocols to prioritize use of diagnostic echocardiography.

## Funding support and author disclosures

Funding to support the clinical study was provided by contract to Mayo Clinic from Anumana, Inc. Dr Carter is a scientific advisor to Anumana, Inc, and has received research funding from Anumana, Inc. Dr Strom is supported by the 10.13039/100000002National Institutes of Health (1R01HL169517, 1K23HL144907, R01AG063937), Ultromics, HeartSciences, Anumana, Philips Healthcare, and EchoIQ. Unrelated to this work, Dr Strom serves on the Scientific Advisory Board for EchoIQ, has received consulting fees from Edwards Lifesciences, Bracco Diagnostics, General Electric Healthcare, and Philips Healthcare; and has served on the data safety monitoring board for Pfizer. Dr Waks is a consultant for Heartbeam Inc; was previously on the scientific advisory board for HeartcoR Solutions, where he remains a consultant; and has received research support from Anumana. Dr Attia is a co-inventor of AI-based algorithms that Mayo Clinic has licensed to Anumana, Inc, with potential for commercialization. Dr Lopez-Jimenez is a member of scientific advisory boards for Anumana, Novo Nordisk, Ionis, New Amsterdam, K-Health, Kento Health, and Wizehealth; is a paid consultant for Regeneron, Novartis, Mediwhale; is an author of a chapter for Up-to-Date; has research supported by Select Research, DMI; and is a co-inventor of the algorithm used in this paper. The algorithm has been licensed to Anumana. Mayo Clinic and other co-inventors may benefit financially if the algorithm becomes commercialized in the future. Dr Friedman is a co-inventor of a number of AI ECG algorithms that have been licensed by Mayo Clinic to Anumana, Eko Health, and AliveCor, and Mayo Clinic. Dr Friedman and other inventors may benefit financially from their commercialization. Dr Awasthi is an employee of Anumana, Inc. Dr Ranganathan is an employee of Anumana, Inc. Dr Barve is an employee of Anumana, Inc. Dr Alger is an employee of Anumana, Inc. Dr Siontis is a co-inventor of AI-based algorithms that Mayo Clinic has licensed to Anumana, Inc, with potential for commercialization; and has received research funding from Anumana, Inc. Dr Noseworthy has an Anumana - License of AI-ECG for AF risk stratification. All other authors have reported that they have no relationships relevant to the contents of this paper to disclose.
