# Supplementary material for: Multisite, External Validation of an AI-Enabled ECG Algorithm for Detection of Low Ejection Fraction
Source: JACC Adv. 2026 Jan 16;5(2):102537. doi: 10.1016/j.jacadv.2025.102537 (PMC12834901; doi:10.1016/j.jacadv.2025.102537)
Supplement: Supplementary Tables 1 to 4 and Supplementary Figures 1 to 6 [file mmc1.docx]

**Supplemental Tables and Figures: Multisite, External Validation of an AI-Enabled ECG Algorithm for Detection of Low Ejection Fraction**

Carter RE, Johnson PW, Strom JB, et al.

Table of Contents

[Supplemental Table 1. Clinical Characteristics by LVEF Classification 2](#_Toc216347424)

[Supplemental Table 2. Intention to Diagnose Analysis 4](#_Toc216347425)

[Supplemental Table 3. Clinical Characteristics by Model Prediction Classification 5](#_Toc216347426)

[Supplemental Table 4. Diagnostic Performance by Site 8](#_Toc216347427)

[Supplemental Figure 1. Device overview 9](#_Toc216347428)

[Supplemental Figure 2. Forest plot of model performance by sex and age group. 10](#_Toc216347429)

[Supplemental Figure 3. Forest plot of model performance by race. 11](#_Toc216347430)

[Supplemental Figure 4. Forest plot of model performance by medical history. 12](#_Toc216347431)

[Supplemental Figure 5. Forest plot of model performance by ECG diagnosis of conduction disorder (n=9,992). 13](#_Toc216347432)

[Supplemental Figure 6. Forest plot of model performance by ECG device. 14](#_Toc216347433)

# Supplemental Table 1. Clinical Characteristics by LVEF Classification

|  | **LVEF > 40% (N=12864)** | **LVEF <= 40% (N=1096)** | **p value** ^a^ |
| --- | --- | --- | --- |
| **Site** |  |  | < 0.001 |
| BIDMC | 3585 (27.9%) | 383 (34.9%) |  |
| MONTEFIORE | 2566 (19.9%) | 151 (13.8%) |  |
| MONUMENT | 3553 (27.6%) | 320 (29.2%) |  |
| UTAH | 3160 (24.6%) | 242 (22.1%) |  |
| **LVEF** | 62.3 (40.1, 93.0) | 30.6 (5.4, 40.0) | < 0.001 |
| **Age (yrs)** | 65.7 (18.0, 105.0) | 70.0 (18.5, 100.0) | < 0.001 |
| **Age Group** |  |  | < 0.001 |
| < 40 | 1568 (12.2%) | 51 (4.7%) |  |
| 40 - 49 | 1204 (9.4%) | 76 (6.9%) |  |
| 50 - 59 | 2027 (15.8%) | 172 (15.7%) |  |
| 60 - 69 | 2938 (22.8%) | 250 (22.8%) |  |
| 70 - 79 | 2742 (21.3%) | 277 (25.3%) |  |
| 80 or more years | 2385 (18.5%) | 270 (24.6%) |  |
| **Sex (male)** | 6548 (50.9%) | 703 (64.1%) | < 0.001 |
| **Height (m)** | 1.7 (1.0, 2.6) | 1.7 (1.2, 2.2) | < 0.001 |
| **Weight (kg)** | 80.6 (12.7, 246.5) | 79.9 (32.6, 199.0) | 0.046 |
| **BMI** | 27.9 (4.3, 111.5) | 27.2 (12.9, 57.0) | < 0.001 |
| **Weight Status** |  |  | < 0.001 |
| Underweight | 369 (3.3%) | 53 (5.2%) |  |
| Normal Weight | 3147 (27.8%) | 310 (30.6%) |  |
| Overweight | 3578 (31.6%) | 329 (32.5%) |  |
| Obese | 4222 (37.3%) | 320 (31.6%) |  |
| **White** | 8258 (66.1%) | 686 (63.9%) | 0.148 |
| **Black or African American** | 1252 (10.0%) | 119 (11.1%) | 0.266 |
| **American Indian or Alaska Native** | 644 (5.2%) | 72 (6.7%) | 0.029 |
| **Asian** | 280 (2.2%) | 20 (1.9%) | 0.419 |
| **Native Hawaiian or other Pacific Islander** | 47 (0.4%) | 8 (0.7%) | 0.068 |
| **Hispanic** | 1387 (11.2%) | 88 (8.5%) | 0.007 |
| **Hypertension** | 7579 (58.9%) | 759 (69.3%) | < 0.001 |
| **Heart Failure** | 2597 (20.2%) | 794 (72.4%) | < 0.001 |
| **Coronary Revascularization** | 1170 (9.1%) | 270 (24.6%) | < 0.001 |
| **Myocardial Infarction** | 2014 (15.7%) | 436 (39.8%) | < 0.001 |
| **Alcoholism** | 1084 (8.4%) | 94 (8.6%) | 0.864 |
| **Diabetes Mellitus** | 3395 (26.4%) | 398 (36.3%) | < 0.001 |
| **Rheumatic Fever** | 5 (0.0%) | 0 (0.0%) | 0.514 |
| **Days from ECG to ECHO** | 1.0 (0.0, 30.0) | 1.0 (0.0, 30.0) | < 0.001 |
| **Conduction Disorder** | 1322 (14.2%) | 196 (27.5%) | < 0.001 |
| Right bundle branch block (RBBB) | 632 (6.8%) | 49 (6.9%) | 0.950 |
| Left bundled branch block (LBBB) | 249 (2.7%) | 105 (14.7%) | < 0.001 |
| Left posterior fascicular block (LPFB) | 30 (0.3%) | 5 (0.7%) | 0.100 |
| Left anterior fascicular block (LAFB) | 221 (2.4%) | 18 (2.5%) | 0.810 |
| Bifascicular block (BFB) | 190 (2.0%) | 19 (2.7%) | 0.267 |

Data presented are mean (min, max) or frequency (percentage). P-values are from a Pearson chi-square test or a two-sample t-test. No p-value is relevant for the comparison of mean LVEF between the dichotomized LVEF levels.

^a^ Missing data are excluded from all calculations. The following is a summary of the missing data for the overall variable along with the sample size by LVEF grouping (LVEF >40:LVEF<=40):Sex (Nmiss = 3 [3:0]), Height (Nmiss = 1,578 [1,496:82]), Weight (Nmiss = 1,093 [1,051:42]), BMI (Nmiss = 1,632 [1,548:84]), Weight Status (Nmiss = 1,632 [1,548:84]), White (Nmiss = 384 [362:22]), Black or African American (Nmiss = 384 [362:22]), American Indian or Alaska Native (Nmiss = 384 [362:22]), Asian (Nmiss = 384 [362:22]), Native Hawaiian or other Pacific Islander (Nmiss = 384 [362:22]), Hispanic (Nmiss = 591 [529:62]), Conduction Disorder (Nmiss = 3,968 [3,585:383]), Right bundle branch block (Nmiss = 3,968 [3,585:383]), Left bundled branch block (Nmiss = 3,968 [3,585:383]), Left posterior fascicular block (Nmiss = 3,968 [3,585:383]), Left anterior fascicular block (Nmiss = 3,968 [3,585:383]) and Bifascicular block (Nmiss = 3,968 [3,585:383]).

# Supplemental Table 2. Intention to Diagnose Analysis

For this analysis, any patient record that did not yield an AI prediction (e.g., due to error) were conservatively coded. Specifically, any true LVEF <= 40% with an error prediction was recoded to be test negative (which penalizes sensitivity). Similarly, LVEF > 40% with an error prediction was coded as a test positive. The following tables give the breakdown of the recoding.

|  | **LVEF > 40% (N=14790)** | **LVEF <= 40% (N=1210)** | **Total (N=16000)** | **p value** |
| --- | --- | --- | --- | --- |
| **Test results (3x2)** |  |  |  | < 0.001 |
| Test Positive | 2115 (14.3%) | 926 (76.5%) | 3041 (19.0%) |  |
| No ECG Prediction | 1926 (13.0%) | 114 (9.4%) | 2040 (12.8%) |  |
| Test Negative | 10749 (72.7%) | 170 (14.0%) | 10919 (68.2%) |  |
| **Test results (3x2 condensed)** |  |  |  | < 0.001 |
| Test Positive (3x2) | 4041 (27.3%) | 926 (76.5%) | 4967 (31.0%) |  |
| Test Negative (3x2) | 10749 (72.7%) | 284 (23.5%) | 11033 (69.0%) |  |

| **Measure** | **Fraction** | **Estimate (95% CI)** |
| --- | --- | --- |
| Sensitivity | 926/1,210 | 76.5% (74.0%, 78.9%) |
| Specificity | 10,749/14,790 | 72.7% (72.0%, 73.4%) |
| Positive Predictive Value | 926/4,967 | 18.6% (17.6%, 19.8%) |
| Negative Predictive Value | 10,749/11,033 | 97.4% (97.1%, 97.7%) |
| LRT+ |  | 2.80 (2.69, 2.92) |
| LRT- |  | 0.32 (0.29, 0.36) |
| Prevalence | 1,210/16,000 | 7.6% (7.2%, 8.0%) |

# Supplemental Table 3. Clinical Characteristics by Model Prediction Classification

| Group | N | True Positives | True Negatives | False Positives | False Negatives |
| --- | --- | --- | --- | --- | --- |
| **Site** |  |  |  |  |  |
| BIDMC | 3,968 | 311 | 3,073 | 512 | 72 |
| MONTEFIORE | 2,717 | 129 | 2,142 | 424 | 22 |
| MONUMENT | 3,873 | 276 | 2,852 | 701 | 44 |
| UTAH | 3,402 | 210 | 2,682 | 478 | 32 |
| **Sex** |  |  |  |  |  |
| Male | 7,251 | 606 | 5,265 | 1,283 | 97 |
| Female | 6,706 | 320 | 5,481 | 832 | 73 |
| Missing | 3 | 0 | 3 | 0 | 0 |
| **Age Group** |  |  |  |  |  |
| < 40 | 1,619 | 34 | 1,455 | 113 | 17 |
| 40 - 49 | 1,280 | 63 | 1,077 | 127 | 13 |
| 50 - 59 | 2,199 | 151 | 1,739 | 288 | 21 |
| 60 - 69 | 3,188 | 215 | 2,466 | 472 | 35 |
| 70 - 79 | 3,019 | 235 | 2,222 | 520 | 42 |
| 80 or more years | 2,655 | 228 | 1,790 | 595 | 42 |
| **White Race** |  |  |  |  |  |
| White | 8,944 | 578 | 6,901 | 1,357 | 108 |
| Other | 4,632 | 329 | 3,545 | 699 | 59 |
| Missing | 384 | 19 | 303 | 59 | 3 |
| **Black Race** |  |  |  |  |  |
| Black | 1,371 | 101 | 1,016 | 236 | 18 |
| Other | 12,205 | 806 | 9,430 | 1,820 | 149 |
| Missing | 384 | 19 | 303 | 59 | 3 |
| **American Indian Race** |  |  |  |  |  |
| American Indian | 716 | 67 | 509 | 135 | 5 |
| Other | 12,860 | 840 | 9,937 | 1,921 | 162 |
| Missing | 384 | 19 | 303 | 59 | 3 |
| **Asian Race** |  |  |  |  |  |
| Asian | 300 | 14 | 256 | 24 | 6 |
| Other | 13,276 | 893 | 10,190 | 2,032 | 161 |
| Missing | 384 | 19 | 303 | 59 | 3 |
| **Native Hawaiian Race** |  |  |  |  |  |
| Native Hawaiian | 55 | 7 | 40 | 7 | 1 |
| Other | 13,521 | 900 | 10,406 | 2,049 | 166 |
| Missing | 384 | 19 | 303 | 59 | 3 |
| **Hispanic Ethnicity** |  |  |  |  |  |
| Hispanic | 1,475 | 77 | 1,198 | 189 | 11 |
| Other | 11,894 | 798 | 9,126 | 1,822 | 148 |
| Missing | 591 | 51 | 425 | 104 | 11 |
| **Weight Status** |  |  |  |  |  |
| Underweight | 422 | 42 | 286 | 83 | 11 |
| Normal Weight | 3,457 | 253 | 2,617 | 530 | 57 |
| Overweight | 3,907 | 287 | 2,997 | 581 | 42 |
| Obese | 4,542 | 277 | 3,529 | 693 | 43 |
| Missing | 1,632 | 67 | 1,320 | 228 | 17 |
| **Hypertension** |  |  |  |  |  |
| Present | 8,338 | 655 | 6,090 | 1,489 | 104 |
| Absent | 5,622 | 271 | 4,659 | 626 | 66 |
| **Heart Failure** |  |  |  |  |  |
| Present | 3,391 | 692 | 1,691 | 906 | 102 |
| Absent | 10,569 | 234 | 9,058 | 1,209 | 68 |
| **Coronary Revascularization** |  |  |  |  |  |
| Present | 1,440 | 243 | 784 | 386 | 27 |
| Absent | 12,520 | 683 | 9,965 | 1,729 | 143 |
| **Myocardial Infarction** |  |  |  |  |  |
| Present | 2,450 | 382 | 1,404 | 610 | 54 |
| Absent | 11,510 | 544 | 9,345 | 1,505 | 116 |
| **Alcoholism** |  |  |  |  |  |
| Present | 1,178 | 83 | 901 | 183 | 11 |
| Absent | 12,782 | 843 | 9,848 | 1,932 | 159 |
| **Diabetes Mellitus** |  |  |  |  |  |
| Present | 3,793 | 356 | 2,638 | 757 | 42 |
| Absent | 10,167 | 570 | 8,111 | 1,358 | 128 |
| **Conduction Disorder** |  |  |  |  |  |
| No | 8,474 | 429 | 6,789 | 1,168 | 88 |
| Yes | 1,518 | 186 | 887 | 435 | 10 |
| Missing | 3,968 | 311 | 3,073 | 512 | 72 |
| **Right bundle branch block (RBBB)** |  |  |  |  |  |
| No | 9,311 | 571 | 7,176 | 1,471 | 93 |
| Yes | 681 | 44 | 500 | 132 | 5 |
| Missing | 3,968 | 311 | 3,073 | 512 | 72 |
| **Left bundled branch block (LBBB)** |  |  |  |  |  |
| No | 9,638 | 513 | 7,614 | 1,416 | 95 |
| Yes | 354 | 102 | 62 | 187 | 3 |
| Missing | 3,968 | 311 | 3,073 | 512 | 72 |
| **Left posterior fascicular block (LPFB)** |  |  |  |  |  |
| No | 9,957 | 610 | 7,657 | 1,592 | 98 |
| Yes | 35 | 5 | 19 | 11 | 0 |
| Missing | 3,968 | 311 | 3,073 | 512 | 72 |
| **Left anterior fascicular block (LAFB)** |  |  |  |  |  |
| No | 9,753 | 598 | 7,504 | 1,554 | 97 |
| Yes | 239 | 17 | 172 | 49 | 1 |
| Missing | 3,968 | 311 | 3,073 | 512 | 72 |
| **Bifascicular block (BFB)** |  |  |  |  |  |
| No | 9,783 | 597 | 7,542 | 1,547 | 97 |
| Yes | 209 | 18 | 134 | 56 | 1 |
| Missing | 3,968 | 311 | 3,073 | 512 | 72 |
| **ECG Source** |  |  |  |  |  |
| GE Healthcare | 10,087 | 650 | 7,897 | 1,414 | 126 |
| Philips Medical Products | 3,873 | 276 | 2,852 | 701 | 44 |
| **Device Model** |  |  |  |  |  |
| D3K | 144 | 2 | 110 | 32 | 0 |
| MAC55 | 5,501 | 322 | 4,312 | 818 | 49 |
| MV360 | 227 | 8 | 183 | 33 | 3 |
| NA | 3,968 | 311 | 3,073 | 512 | 72 |
| Other system | 247 | 7 | 219 | 19 | 2 |
| Phillips 860315 | 3,873 | 276 | 2,852 | 701 | 44 |
| **Days from ECG to Echo** |  |  |  |  |  |
| same day | 1,837 | 187 | 1,311 | 299 | 40 |
| 1-3 days | 8,578 | 561 | 6,493 | 1,433 | 91 |
| 3-7 days | 1,212 | 61 | 965 | 168 | 18 |
| 7-14 days | 887 | 43 | 746 | 88 | 10 |
| 14+ days | 1,446 | 74 | 1,234 | 127 | 11 |

# Supplemental Table 4. Diagnostic Performance by Site

| **Site** | **Measure** | **Fraction** | **Estimate (95% CI)** |
| --- | --- | --- | --- |
| BIDMC | Sensitivity | 311/383 | 81.2% (76.9%, 85.0%) |
|  | Specificity | 3,073/3,585 | 85.7% (84.5%, 86.8%) |
|  | Positive Predictive Value | 311/823 | 37.8% (34.5%, 41.2%) |
|  | Negative Predictive Value | 3,073/3,145 | 97.7% (97.1%, 98.2%) |
|  | LRT+ |  | 5.69 (5.18, 6.24) |
|  | LRT- |  | 0.22 (0.18, 0.27) |
|  | Prevalence | 383/3,968 | 9.7% (8.8%, 10.6%) |
|  |  |  |  |
| MONTEFIORE | Sensitivity | 129/151 | 85.4% (78.8%, 90.6%) |
|  | Specificity | 2,142/2,566 | 83.5% (82.0%, 84.9%) |
|  | Positive Predictive Value | 129/553 | 23.3% (19.9%, 27.1%) |
|  | Negative Predictive Value | 2,142/2,164 | 99.0% (98.5%, 99.4%) |
|  | LRT+ |  | 5.17 (4.64, 5.77) |
|  | LRT- |  | 0.17 (0.12, 0.26) |
|  | Prevalence | 151/2,717 | 5.6% (4.7%, 6.5%) |
|  |  |  |  |
| MONUMENT | Sensitivity | 276/320 | 86.2% (82.0%, 89.8%) |
|  | Specificity | 2,852/3,553 | 80.3% (78.9%, 81.6%) |
|  | Positive Predictive Value | 276/977 | 28.2% (25.4%, 31.2%) |
|  | Negative Predictive Value | 2,852/2,896 | 98.5% (98.0%, 98.9%) |
|  | LRT+ |  | 4.37 (4.04, 4.73) |
|  | LRT- |  | 0.17 (0.13, 0.23) |
|  | Prevalence | 320/3,873 | 8.3% (7.4%, 9.2%) |
|  |  |  |  |
| UTAH | Sensitivity | 210/242 | 86.8% (81.8%, 90.8%) |
|  | Specificity | 2,682/3,160 | 84.9% (83.6%, 86.1%) |
|  | Positive Predictive Value | 210/688 | 30.5% (27.1%, 34.1%) |
|  | Negative Predictive Value | 2,682/2,714 | 98.8% (98.3%, 99.2%) |
|  | LRT+ |  | 5.74 (5.21, 6.32) |
|  | LRT- |  | 0.16 (0.11, 0.22) |
|  | Prevalence | 242/3,402 | 7.1% (6.3%, 8.0%) |
| Overall | Breslow-Day |  | p=0.45 |

# Supplemental Figure 1. Device overview

# Supplemental Figure 2. Forest plot of model performance by sex and age group.

# Supplemental Figure 3. Forest plot of model performance by race.

The figure includes the records that were not extracted with a race classification. The p-values included are the Breslow-Day test of homogeneity of the odds ratio.

# Supplemental Figure 4. Forest plot of model performance by medical history.

The p-values included are the Breslow-Day test of homogeneity of the odds ratio.

# Supplemental Figure 5. Forest plot of model performance by ECG diagnosis of conduction disorder (n=9,992).

The p-values included are the Breslow-Day test of homogeneity of the odds ratio. One site did not supply the necessary XML diagnosis nodes to determine the conduction disorder status (n=3,968). The classification of conduction disorder is the presence of any of the five specific conditions listed below.

# Supplemental Figure 6. Forest plot of model performance by ECG device.

The p-values included are the Breslow-Day test of homogeneity of the odds ratio. One site did not supply the necessary XML header files to determine the model number of the GE ECG machine used to acquire the ECG (coded as NA for the device model). The “other system” class consists of GE S8500 (n=3), GE MAC5k (n=5), GE MAC16 (n=126) and GE MAC2k (n=113).
